# Fusaricidin-Type Compounds Create Pores in Mitochondrial and Plasma Membranes of Mammalian Cells

**DOI:** 10.3390/biom9090433

**Published:** 2019-09-01

**Authors:** Raimo Mikkola, Maria Andersson, Ekaterina Kharechkina, Svetlana Kruglova, Alexey Kruglov

**Affiliations:** 1 Department of Civil Engineering, School of Engineering, Aalto University, 00076 Aalto, Finland; 2 Institute of Theoretical and Experimental Biophysics, Russian Academy of Sciences, Pushchino, 142292 Moscow Region, Russia; 3 Institute of Basic Biological Problems, Russian Academy of Sciences, Pushchino, 142292 Moscow Region, Russia

**Keywords:** fusaricidin, LI-F compounds, mitochondria, plasma membrane, pore, alamethicin, permeability transition pore

## Abstract

Fusaricidins and related LI-F compounds are effective bactericides and fungicides. Recently, we have found that they are highly toxic to mammalian cells. Here, we studied the effect of fusaricidin-type compounds (FTCs) on the membranes of mammalian cells. Ethanol extracts from *Paenibacillus polymyxa* strains, RS10 and I/Sim, were fractionated and analyzed by HPLC and mass spectrometry. The effects of FTCs on mitochondrial functions and integrity were studied by standard methods: measurements of swelling, membrane potential (ΔΨ_m_), respiration rate, cytochrome c release, and pore sizes. Superoxide flashes were registered by 3,7-dihydro-2-methyl-6-(4-methoxyphenyl)imidazol[1,2-a]pyrazine-3-one (MCLA). Plasma membrane permeability was assessed by propidium iodide (PI) staining and ATP release. FTCs caused the permeabilization of the inner mitochondria membrane (IMM) to ions and low-molecular-weight (~750 Da) solutes. The permeabilization did not depend on the permeability transition pore (mPTP) but was strongly dependent on ΔΨ_m_. Fusaricidins A plus B, LI-F05a, and LI-F05b–LI-F07b permeabilized IMM with comparable efficiency. They created pores and affected mitochondrial functions and integrity similarly to mPTP opening. They permeabilized the sperm cell plasma membrane to ATP and PI. Thus, the formation of pores in polarized membranes underlays the toxicity of FTCs to mammals. Besides, FTCs appeared to be superior reference compounds for mPTP studies.

## 1. Introduction

Fusaricidin-type compounds (FTCs) (fusaricidins A–D and LI-Fs) produced by *Paenibacillus polymyxa* is a group of closely related lipodepsipeptides with a microcyclic ring consisting of six amino acid residues linked to the 15-guanidino-3-hydroxypentadecanoic acid tail [1,2,3,4,5]. They effectively kill various Gram-positive bacteria (*Micrococcus*, *Staphylococcus*, and *Bacillus*), fungi (*Aspergillus*, *Fusarium*, and *Penicillium*), and yeast (*Saccharomyces cerevisiae*) at concentrations of 0.8–16 µg/mL [6,7]. FTCs are active against phytopathogens *Leptosphaeria maculans* [8] and *Phytophthora* [9].

It has been established earlier that the intraperitoneal injection of different FTCs induces moderate mortality of mice [1]. Recently, we demonstrated that a bedding material contaminated with FTCs-producing *P. polymyxa* strains caused the death of piglets. The autopsy of piglets revealed hemorrhages in the heart muscle. A crude ethanol extract of the bedding dust was extremely toxic in the boar sperm motility inhibition assay; the EC50 concentration was 0.5 µg/mL. Also, it was found that long-term exposure of employees of one taxi station in western Finland to fusaricidin-containing dust harmed their health [10].

The toxicity of fusaricidins and LI-F compounds to mammalian cells was associated with the damage to mitochondria and plasma membrane and the activation of apoptosis [10]. Because FTCs formed channels permeable to potassium in black lipid membranes, we suggested that their toxicity might be connected with the valinomycin- or cereulide-like ionophoric effect [11]. However, other mechanisms, including the regulation of permeability transition pore (mPTP), cannot be excluded. Indeed, mPTP, a nonselective channel of unknown molecular nature [12] in the inner mitochondrial membrane, becomes permeable for ions and solutes (≤1500 Da) after activation/opening by Ca^2+^ (and numerous additional regulators) [13]. Its opening triggers the high-amplitude mitochondrial swelling, the release of apoptogenic factors, and cell death [14]. Earlier, it was established that cationic peptides BTM-P1 [15], p13II [16], and mastoparan [17] stimulated the mPTP opening. FTCs, being lipopeptides with the guanidine moiety, which bears a positive charge at neutral pH, may participate in mPTP regulation.

Here, we presented the results of a detailed study of the effects of FTCs on mitochondrial and plasma membranes. We found that fusaricidins and LI-Fs made inner mitochondrial and plasma membranes permeable for ions and low-molecular-weight solutes by creating nonselective pores. These pores were somewhat smaller than the mPTP and produced similar effects on mitochondrial respiration, swelling, superoxide flashes, and the release of cytochrome *c*. Our results demonstrated that FTCs could be a better substitution for alamethicin in studies of mitochondrial pathophysiologic states, such as mPTP opening.

## 2. Materials and Methods

### 2.1. Reagents

5,5′,6,6′-tetrachloro-1,1′,3,3″-tetraethylbenzimidazolylcarbocyanine iodide (JC-1), calcein-AM, propidium iodide (PI), and rhodamine 123 were from Invitrogen (Carlsbad, CA, USA). The ATP Biomass Kit HS was from BioThema AB (Haninge, Sweden). Alamethicin A4665, ascorbic acid, bovine serum albumin (BSA), carbonyl cyanide p-(trifluoromethoxy)phenyl-hydrazone (FCCP), cyclosporin A (CsA), 7- dihydro-2-methyl-6-(4-methoxyphenyl)imidazol[1,2-a]pyrazine- 3-one (MCLA), dithionite, ethylene glycol-bis(2-aminoethylether)-N,N,N′,N′-tetraacetic acid (EGTA), glutamate, 4-(2-hydroxyethyl)piperazine-1-ethanesulfonic acid (HEPES), mannitol, malate, polymyxin B sulfate, rotenone, sucrose, succinate, Trizma Base, tetraphenylphosphonium chloride (TPP+), N,N,N’N’-tetramethyl-p-phenylenediamide (TMPD), valinomycin, and polyethyleneglycols of different molecular weights were obtained from the Sigma-Aldrich Corporation (St Louis, MO, USA). Other chemicals were of analytical grade and were purchased from local suppliers.

### 2.2. Sperm Cells

The extended boar semen (27×10^6^ cells/mL) was a commercial product for pig breeding from Figen Ltd (Tuomikylä, Finland). Semen was used for experiments within two days after collection. The semen batch was rejected if less than 80% of sperm cells were motile.

### 2.3. Paenibacillus Polymyxa Strains

Pure cultures of *P. polymyxa* strains, RS10 (HAMBI 3103) and I/Sim (HAMBI 3104), which are toxic to mammalian cells, were obtained and identified, as described previously [10,18], and were deposited in the HAMBI culture collection (University of Helsinki, Faculty of Agriculture and Forestry, Division of Microbiology and Biotechnology).

### 2.4. HPLC and Mass Spectrometry Analysis

The ethanol extracts of the *P. polymyxa* strains, RS10 and I/Sim, were fractionated by RP-HPLC using 1100 series LC (Agilent Technology, Wilmington, DE, USA) and an Atlantis column C18 T3 4.6 i. d. × 150 mm, 3 μm (Waters, Milford, MA, USA). The eluents were 0.1% formic acid (A) and methanol (B). Compounds were separated using isocratic elution with 80% B and 20% A for 20 min at a flow rate of 1 mL/min. For detection, absorbance at 215, 240, 254, and 280 nm was measured. Fractions were collected once a minute. Electrospray ionization-ion trap mass spectrometry analysis of ethanol extracts of the *P. polymyxa* strains, RS10 and I/Sim, was performed using an MSD-Trap-XCT_plus ion trap mass spectrometer equipped with an Agilent ESI source and Agilent 1100 series LC (HPLC-ESI-IT-MS) (Agilent Technologies, Wilmington, DE, USA). HPLC-ESI-IT-MS was performed using the positive mode in a range of 50–2000 *m*/*z*. The conditions of HPLC of samples were as described above.

### 2.5. Isolation and Purification of Rat Liver Mitochondria

All manipulations with animals before the isolation of the liver were performed per the Helsinki Declaration of 1975 (revised in 1983), national requirements for the care and use of laboratory animals, and protocol 25/219 (of 26.04.2019) approved by the Commission on Biological Safety and Bioethics of the Iinstitute of Theoretical and Experimental Biophysics, Russian Academy of Sciences. Adult male Wistar rats were killed by cutting the neck after anesthesia with CO_2_. Rat liver mitochondria were isolated according to a standard differential centrifugation procedure [19]. The homogenization medium contained 220 mM mannitol, 70 mM sucrose, 10 mM HEPES (pH adjusted to 7.4 with Trizma Base), 1 mM EGTA, and 0.05% BSA. The pellet was washed two times with the same medium but without EGTA and BSA. Final pellets were resuspended in this medium to yield 70–80 mg protein/mL. Measurements were performed at the ambient temperature (22 ± 2 ºC) using the standard KCl-based medium (125 mM KCl, 20 mM mannitol, 10 mM HEPES (pH 7.3), 2 mM KH_2_PO_4_) unless otherwise indicated. Other experimental details are given in figures and figure legends. The total mitochondrial protein was determined by the Biuret method using BSA as a standard [20].

### 2.6. Recording of the Permeabilization of Mitochondrial Membranes

The permeabilization of mitochondrial membranes for solutes was assessed by high-amplitude mitochondrial swelling. Swelling was defined as a decrease in A_540_ (Uvikon 923 spectrophotometer (Kontron Instruments, USA)) or A_550_ (Infinite 200 plate reader (Tecan, Austria)) in different incubation media: KCl-based medium, NaCl-based medium (125 mM NaCl, 20 mM mannitol, 10 mM HEPES (pH 7.3), 2 mM NaH_2_PO_4_), and in inorganic phosphate-free sucrose/mannitol-based medium (60 mM sucrose, 200 mM mannitol, 10 mM HEPES (pH 7.3)). Media also contained respiratory substrates and other additions, which is specified in figure legends.

### 2.7. Assessment of Membrane Potential (ΔΨm)

The measurements of ΔΨ_m_ across the inner mitochondrial membrane were performed in a standard medium supplemented with respiratory substrates and 330 nM rhodamine 123 using a plate reader Infinite 200. Alternatively, ΔΨ_m_ was assessed by measuring the distribution of TPP^+^ between mitochondria and the solution using a TPP^+^-selective electrode (Niko Analyt, Moscow, Russia) connected to a multielectrode system Record 4 (Institute of Theoretical and Experimental Biophysics, RAS, Russia).

### 2.8. Measurements of the Rate of Oxygen Consumption by Isolated Mitochondria

The mitochondrial respiration was recorded in a closed thermostated chamber equipped with a magnetic stirrer using a Clark-type oxygen electrode (a workshop of the Institute of Theoretical and Experimental Biophysics, RAS, Russia) and a computerized system Record 4 simultaneously with the registration of ΔΨ_m_. Electrodes were calibrated before each experiment per the guidance of the program designed by Record 4 manufacturers. All additions were made through a small hole in the cap of the chamber.

### 2.9. Registration of Superoxide Anion Production

The rate of superoxide anion production was assessed using the highly sensitive chemiluminescent probe MCLA (3,7-dihydro-2-methyl-6-(4-methoxyphenyl)imidazol[1,2-a]pyrazine-3-one) [21]. The kinetics of MCLA-derived chemiluminescence (MDCL) was recorded using a plate reader (Infinite 200 Tecan, Austria), as described earlier [22]. Each value on the curve is the mean ± S.E.M. of three integrations of luminescence for 900 ms expressed in arbitrary units.

### 2.10. Measurement of the Release of Cytochrome C From Mitochondria

The release of cytochrome *c* was measured, as described earlier [23]. Mitochondria (2 mg/mL) were incubated in standard KCl-based medium supplemented with respiratory substrates with continuous control of respiration and ΔΨ_m_. Mitochondrial swelling and the accompanying cytochrome *c* release were initiated by the addition of Ca^2+^, alamethicin, polymyxin B, RS10 extract, and purified fusaricidins A plus B and LI-F05a at indicated concentrations. After 15-min incubation with stirring, mitochondria were sedimented, and the absorption spectra of supernatants (reduced with dithionite and oxidized) were recorded six times using an Uvikon 923 spectrophotometer (Kontron Instruments, San Diego, CA, USA) and averaged. The extinction coefficient for cytochrome *c* (A_550_–A_540_ nm) was taken to be 18.2 mM^−1^∙cm^−1^.

### 2.11. Measurement of the Size of Pores in the Mitochondrial Inner Membrane

The size of pores created by alamethicin and fusaricidins or induced by Ca^2+^ (mPTP) was measured by the solute size-exclusion test [17] with modifications [24]. Mitochondria (1 mg/mL) were exposed to Ca^2+^ (250 nmol/mg protein), alamethicin (12.5 µg/mL), and fusaricidins A plus B (10.5 µg/mL) and allowed to swell for 10 min in standard KCl-based medium. EGTA (1 mM) and 1 µM CsA (cyclosporin A) were added to samples exposed to alamethicin and fusaricidins to prevent spontaneous mPTP opening. After the termination of high-amplitude swelling recorded using an Uvikon 923 spectrophotometer, solutions of polyethylene glycols (PEGs) of different molecular weights (200–8000 Da) were added to create a 40% increase in the osmotic pressure (10 % (*v*/*v*) of PEGs solution). The shrinkage of the mitochondrial matrix was assessed by an increase in absorbance at 540 nm. The complete restoration of the matrix volume was assumed to be caused by PEGs, which are completely incapable of penetrating through the inner membrane.

### 2.12. Assay of the Toxicity of Fusaricidins to Sperm Cells

Toxic compounds were dissolved in 1 µL of ethanol, dispensed in 200 μL of boar semen (in manufacturer’s Extender solution), and examined after 1, 24, and 72 h of incubation using a phase-contrast microscope (with a heated stage) for the motility of spermatozoa, as described by Andersson et al. [25]. The calibration of the bioassay was performed using valinomycin. The integrity of the plasma membrane was assessed by propidium iodide (PI) staining, and changes in ΔΨ_m_ were estimated by staining with JC-1. All tests were run three times. The differences between the results of replicate tests were less than between one dilution step (two-fold).

### 2.13. Measurement of ATP Release from Sperm Cells

The release of ATP from sperm cells was measured using an ATP Biomass Kit HS per the manufacturer’s protocol with the following modifications. Sperm cells were incubated with compounds or lysis buffer (1:1 *v*/*v*) (positive control) for 5 min and then sedimented for 5 min at 15,000 g. Supernatants were then analyzed for the ATP content using a Wallac 1250 luminometer (LKB Instruments Company, Mount Waverley, Australia).

### 2.14. Statistical Analysis

The data presented are either the means ± standard error of means (S.E.M.) or representative data for three or more experiments.

## 3. Results

### 3.1. RS10 and I/Sim Extracts Induce Mitochondrial Swelling

**Figure 1:**
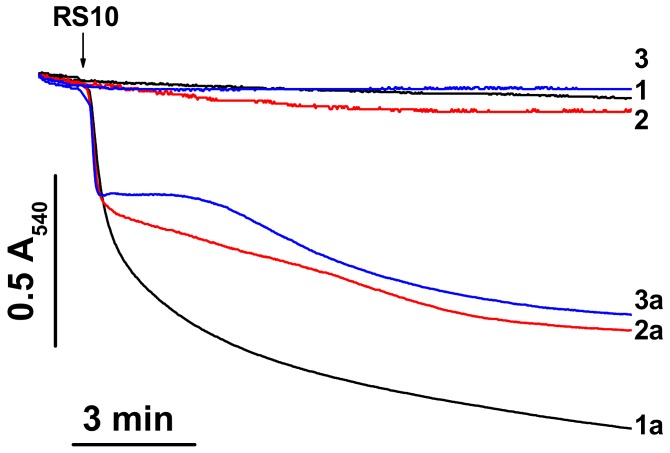
RS10 induces mitochondrial swelling in media of different ionic composition. Just before measurements, mitochondria (0.5 mg protein/mL) were added to sucrose-mannitol- (1, 1a), KCl- (2, 2a), and NaCl-based media (3, 3a) supplemented with 5 mM glutamate and 5 mM malate. The arrow indicates the addition of an RS10 extract (15 µg/mL) (figures with index “a”). Representative data of one experiment of at least three similar are shown. The experiments were performed at 23 °C.

Ethanol extracts of *P. polymyxa* strains, RS10 and I/Sim, and their fusaricidin-containing HPLC fractions caused an increase in the ionic permeability of biological and artificial lipid membranes [10]. However, the ionic selectivity of the permeabilization of biological membranes remained unclear. To address this question, we assessed the effect of RS10 and I/Sim ethanol extract on the mitochondrial swelling in media of different ionic composition. (Selective ionophores and carriers stimulate the swelling of energized mitochondria in media enriched with transported ions.). Figure 1 shows that an RS10 extract caused a high-amplitude swelling of liver mitochondria in sucrose-mannitol-, KCl-, and NaCl-based medium (1a, 2a, and 3a, respectively). The amplitude of swelling in sucrose-mannitol medium (low ionic strength) was greater than in salt-based media. Similar data were obtained for an I/Sim extract (not shown). Thus, RS10 and I/Sim extracts induced nonselective permeabilization of the inner mitochondria membrane (IMM) to ions and low-molecular-weight solutes. Presumably, ionic interactions contribute to the creation and/or stabilization of fusaricidin pores or channels.

### 3.2. RS10 Extract Permeabilizes Mitochondrial Membranes in an mPTP-Independent and ΔΨ_m_-Dependent Manner

**Figure 2:**
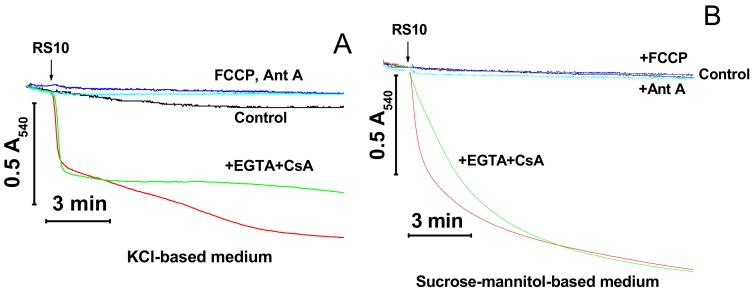
Permeabilization of mitochondrial membranes by FTCs (fusaricidin-type compounds) is membrane potential (ΔΨ_m_)-dependent and permeability transition pore (mPTP)-independent. Immediately before measurements, mitochondria (0.5 mg protein/mL) were added to KCl- (**A**) and sucrose-mannitol-based medium (**B**), supplemented with 5 mM glutamate and 5 mM malate. The arrow indicates the addition of the RS10 extract (15 µg/mL) to all samples except control (black). Where shown, media also contained 0.5 mM EGTA and 2 µM CsA (cyclosporin A) (green), antimycin A (2 µg/mL) (Ant A, blue), and 0.5 µM FCCP (carbonyl cyanide p-(trifluoromethoxy)phenyl-hydrazone) (navy blue). Representative data of one experiment of at least three similar are shown. The experiments were performed at 23 °C.

Cationic peptides are known to facilitate the mPTP opening by Ca^2+^ [15‒17]. We explored whether the inner membrane permeabilization caused by the RS10 extract is brought about through the induction of mPTP opening. Figure 2 shows that the calcium chelator EGTA and the mPTP inhibitor CsA did not prevent the mitochondrial swelling both in KCl- (A) and sucrose–mannitol-based media (B).

By contrast, agents that cause the dissipation of ΔΨ_m_ through different mechanisms, the protonophore FCCP and the inhibitor of respiratory chain complex III antimycin A (Ant A), completely precluded the RS10-dependent swelling in both media. These data indicated that FTCs did not induce the mPTP opening *per se* though they could stimulate pore opening to some extent via the depolarization of the IMM. Besides, ΔΨ_m_ was critical for the permeabilization of mitochondrial membranes by RS10. Presumably, ΔΨ_m_ facilitates the incorporation of positively charged FTCs into the mitochondrial membrane.

### 3.3. Content of Fusaricidin-Type Compounds in RS10 and I/Sim Extracts

**Figure 3:**
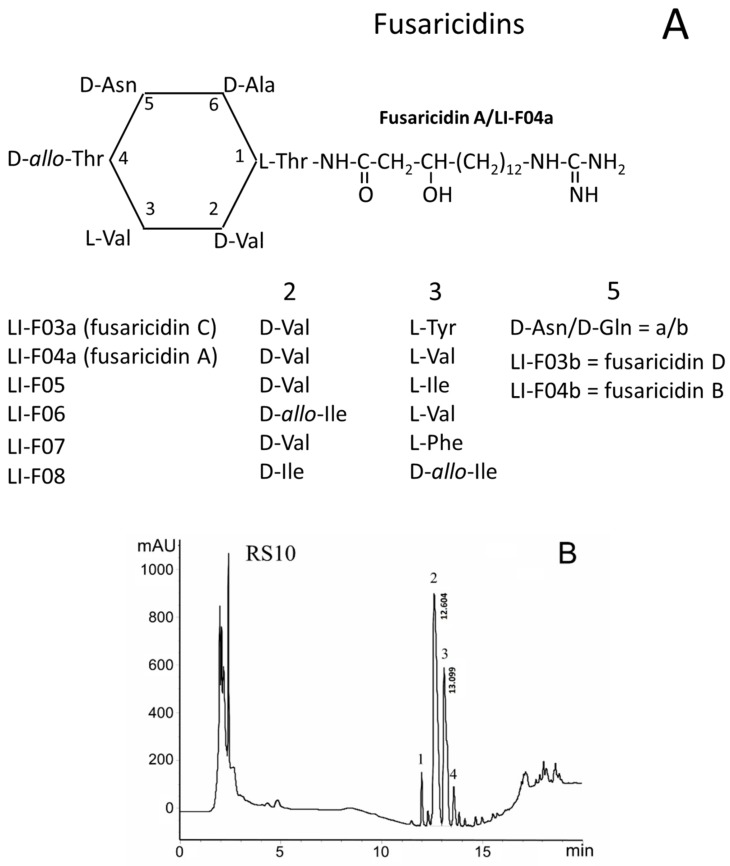

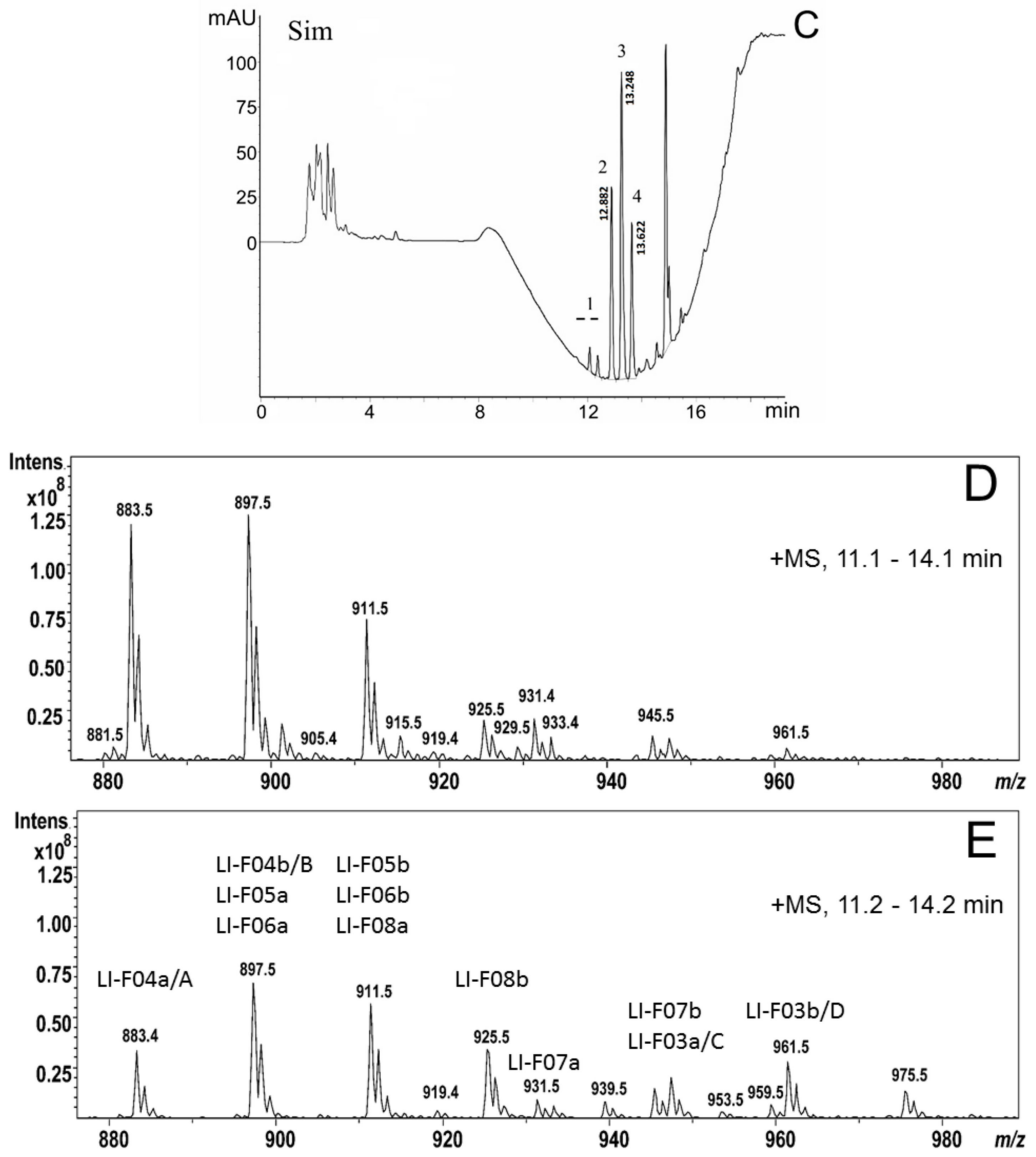
Content of FTCs in RS10 and I/Sim extracts. (**A**) Amino acid substitutions in FTCs. (**B**,**C**) HPLC fractionation of RS10 (**B**) and I/Sim ethanol extracts (**C**) (10 and 1 µL, respectively); numbers 1–4 show toxic fractions in the extracts. (**D**,**E**) Mass spectra of toxic HPLC fractions (range 11–14 min) of RS10 (**D**) and Sim (**E**) extracts. Representative data of at least three runs are shown.

FTCs are closely related cyclic lipodepsipeptides with the ring consisting of six amino acid residues linked to the positively charged 15-guanidino-3-hydroxypentadecanoic acid tail (Figure 3A). FTCs differ in amino acids at positions 2, 3, and 5 in the ring [1,2,3,4]. FTCs with D-asparagine or D-glutamine at the 5th position are designated by the letters *a* and *b*, respectively. The fractionation by HPLC of heat-treated ethanol extracts of *P. polymyxa* strains, RS10 (B) and I/Sim (C), revealed the presence of four fractions of toxic substances, which contained twelve FTCs identified as fusaricidins A–D and LI-Fs (Table 1) [10].

As it follows from the averaged mass spectrum profiles with a retention time range of 11–14 min, the extracts from *P. polymyxa* strains, RS10 (D) and I/Sim (E), contained the same fusaricidins and LI-Fs but in different ratios. A comparison of the data, presented in panels B/D and C/E, allowed one to estimate the relative amount of various FTCs in RS10 and I/Sim extracts. In both extracts, the main components in fractions 1, 2, 3, and 4 were fusaricidins C and D, fusaricidins A and B, LI-F05a, and LI-F05b–LI-F07b, respectively (Table 1) [10].

However, fusaricidins A plus B (fraction 2) and LI-F05a (fraction 3) dominated in RS10 and I/Sim extracts, respectively. Besides, the RS10 extract gave a high yield of LI-F05a (fraction 3), while the I/Sim extract contained a high amount of fusaricidins A plus B (fraction 2) and LI-F05b–LI-F07b (fraction 4). The content of fusaricidins C and D (fraction 1) was low in both extracts. Therefore, for further studies, we used fractions 2 and 3 of the RS10 extract and fractions 2, 3, and 4 of the I/Sim extract.

### 3.4. Mitochondrial Swelling Induced by Purified FTCs from RS10 and I/Sim Extracts

**Figure 4:**
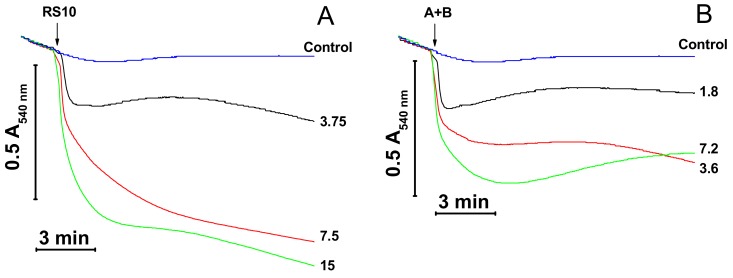

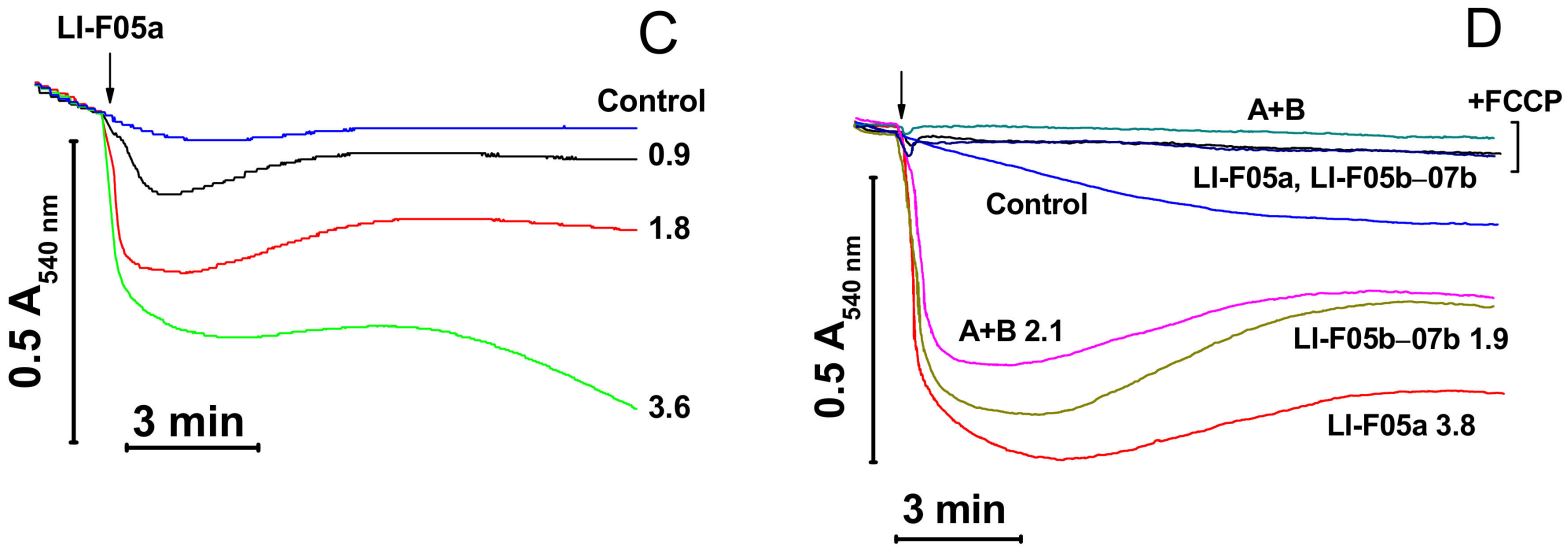
Effect of purified FTCs on the swelling of mitochondria with polarized and depolarized inner membranes. Immediately before measurements, mitochondria (0.5 mg protein/mL) were added to KCl-based medium supplemented with 5 mM glutamate, 5 mM malate, 1 mM EGTA, and, where indicated, 500 nM FCCP (**D**). The arrow shows the addition of RS10 (**A**), fusaricidins A + B (**B**,**D**), LI-F05a (**C**,**D**), and LI-F05b – LI-F07b (**D**) at indicated concentrations (µg/mL) to all samples except control. Representative data of at least three experiments are shown. The experiments were performed at 23 °C.

We examined the effect of fusaricidins A plus B (fraction 2) and LI-F05a (fraction 3) purified from the RS10 extract and fusaricidins A plus B (fraction 2), LI-F05a (fraction 3), and LI-F05b–LI-F07b (fraction 4) purified from the I/Sim extract on the mitochondrial mPTP-independent swelling and its dependence on ΔΨ_m_ (Figure 4). As it follows from Figure 4, purified fusaricidins A+B (panels B and D), LI-F05a (panels C and D), and LI-F05b–LI-F07b (panel D) exhibited similar efficiency in the permeabilization of mitochondrial membranes. Moreover, the permeabilization of the IMM by all FTCs was ΔΨ_m_-dependent since it was completely precluded by the ΔΨ_m_ disruptors FCCP (D) and antimycin A (not shown). (The FTC/FCCP molar ratio in the medium was ~4.3, 8.4, and 4.1 for fusaricidins A+B, LI-F05a, and LI-F05b – LI-F07b, respectively.). Hence, all FTCs tested possessed a comparable pore-forming ability.

### 3.5. Effects of FTCs on Mitochondrial Functions and Integrity

**Figure 5:**
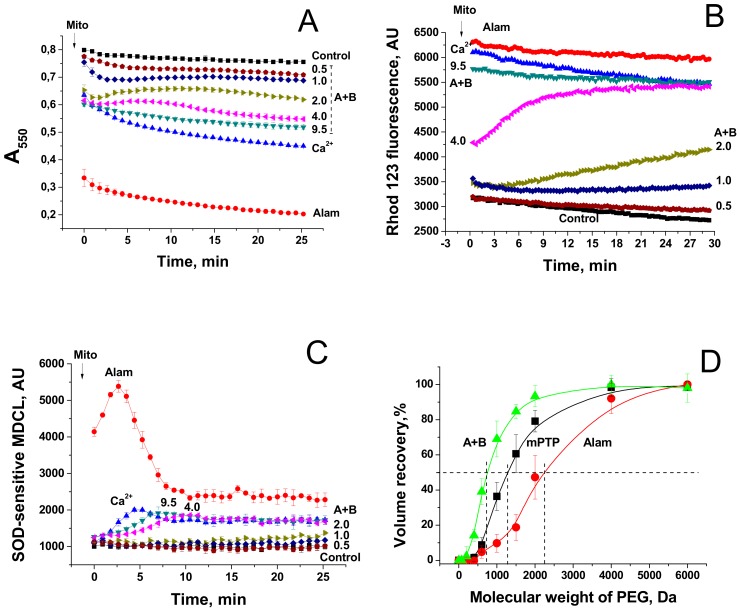
Dynamics of mitochondrial swelling (**A**), ΔΨ_m_ (**B**), and superoxide anion production (**C**) in mitochondria permeabilized by alamethicin, fusaricidins, and the mPTP inductor Ca^2+^. Comparison of the sizes of mPTP and pores created by fusaricidins and alamethicin (**D**). (**A**–**C**) Mitochondria (0.5 mg prot./mL) were added to a standard incubation medium supplemented with 5 mM succinate (plus rotenone 2 µg/mL), 330 nM rhodamine (Rhod) 123 (**B**), and 20 µM MCLA (3,7-dihydro-2-methyl-6-(4-methoxyphenyl)imidazol[1,2-a]pyrazine-3-one) (**C**). The arrow shows when mitochondria (Mito) were transferred to wells by alamethicin (Alam, 20 µg/mL), 200 µM Ca^2+^, and fusaricidins A+B (fraction 2) at indicated concentrations (µg/mL). All samples except those with Ca^2+^ contained 1 mM EGTA. Representative traces of one experiment of at least three identical are shown. The data on panels A and C were recorded synchronously. (**C**) Reference wells also contained superoxide dismutase (SOD, 200 U/mL). The SOD-sensitive part of an MDCL (MCLA-derived chemiluminescence) signal is presented. (**D**) Recovery of mitochondrial volume after the addition of PEG of different molecular weight to mitochondria swollen due to the creation of pores by alamethicin (Alam) (12.5 µg/mL) and fusaricidins A + B (10.5 µg/mL) and the opening of the Ca^2+^-dependent mPTP (250 nmol CaCl_2_/mg protein). The data presented are the means ± S.E.M. (*n* = 3) for three independent measurements. The experiments were performed at 23 °C.

**Figure A1:**
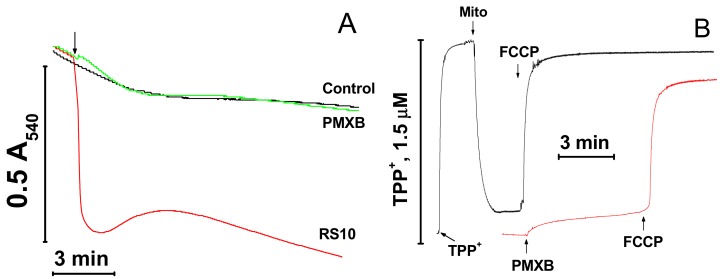
Effect of polymyxin B on mitochondrial swelling (**A**) and ΔΨ_m_ (**B**). (**A**) Mitochondria (0.5 mg prot./mL) were added to a standard KCl-based medium supplemented with 5 mM succinate (plus 2 µg/mL rotenone) immediately before measurements. The arrow indicates the addition of the RS10 extract (10 µg/mL) and polymyxin B (PMXB) (100 µg/mL). (**B**) Arrows show the addition of 1.5 µM TPP^+^ (tetraphenylphosphonium chloride), mitochondria (1.5 mg prot./mL) (Mito), polymyxin B (100 µg/mL) (PMXB), and 500 nM FCCP. The figure shows the original traces of one standard experiment of three identical.

FTCs induce the apoptosis-like death of murine fibroblasts L929 and porcine kidney tubular epithelial cells PK-15 [10]. It is well established that apoptosis can be activated by the release of mitochondrial apoptogenic factors [13,14] and reactive oxygen species [26] after the permeabilization of mitochondrial membranes. Therefore, we compared the effects of the pore-forming peptide alamethicin, lipopeptides FTCs, and polymyxin B and the mPTP inductor Ca^2+^ on mitochondrial functions and integrity. Figure 5A,B indicate that fusaricidins A + B caused the swelling (A) and the dissipation of ΔΨ_m_ (B), which, at high fusaricidin concentrations (9.5 µg/mL), were similar to those induced by mPTP opening. Besides, fusaricidins A+B at high concentrations stimulated the generation of superoxide anion (superoxide dismutase-sensitive MDCL) by mitochondria to the same level as it was stimulated by mPTP opening (C). By contrast, alamethicin induced a much stronger swelling (A) and superoxide flash (C). The fluorescence of rhodamine 123 in a mitochondrial suspension in the presence of alamethicin was also higher (indicating a lower ΔΨ_m_) than in the presence of Ca^2+^ and fusaricidins (B). However, this, at least in part, might be due to the clearance of suspension. Polymyxin B, the other positively charged antibiotic produced by *P. polymyxa*, at high concentrations (≥100 µg/mL) had a minimum effect on the mitochondrial swelling and ΔΨ_m_ (Figure A1) under the same experimental conditions. We assessed the size of pores created by FTCs in the IMM using PEG of different molecular weights. It is seen from Figure 5D that fusaricidins A+B (10.5 µg/mL) created pores permeable for compounds of an average weight of 750 Da. These pores were considerably smaller than permeability transition pores induced by Ca^2+^ (250 nmol/mg protein), ~1350 Da, and pores created by alamethicin (12.5 µg/mL), ~2250 Da. The same pore sizes were measured for LI-F05a and the RS10 extract (not shown).

**Figure 6:**
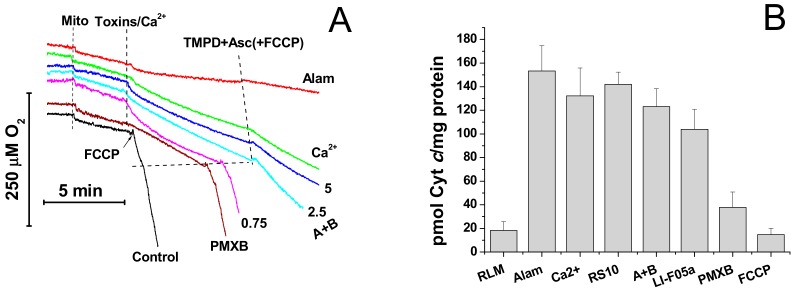
Effects of the pore formers alamethicin, fusaricidins, and polymyxin B and the mPTP inductor Ca^2+^ on mitochondrial respiration (**A**) and the release of cytochrome *c* (**B**). (**A**) Mitochondria (Mito) (1.5 mg prot./mL) were added to the medium with the same composition as in Figure 5A. Where shown, toxins (alamethicin (Alam, 10 µg/mL), fusaricidins A+B (0.75, 2.5, and 5 µg/mL), polymyxin B (PMXB, 100 µg/mL)), 200 µM Ca^2+^, 200 µM TMPD (N,N,N’N’-tetramethyl-p-phenylenediamide), 2 mM ascorbate (Asc), and 500 nM FCCP were added to the suspension. The figure shows the original traces of one standard experiment of three identical. (**B**) Mitochondria (2 mg prot./mL) were incubated in the presence of RS10 (30 µg/mg prot.), fusaricidins A+B (8.4 µg/mg prot.), LI-F05a (4.5 µg/mg prot.), alamethicin (12.5 µg/mg) (Alam), polymyxin B (100 µg/mg protein) (PMXB), 500 nM FCCP, and Ca^2+^ (250 nmol/mg of protein). The release of cytochrome *c* (Cyt *c*) was measured as described in the Materials and Methods Section. The data presented are the means ± S.E.M. for three independent experiments. The experiments were performed at 23 °C.

Then, we assessed the effect of fusaricidins on the respiration rate and the release of cytochrome *c* from mitochondria. It is seen from Figure 6A that alamethicin, Ca^2+^, and fusaricidins A + B at different concentrations transiently activated the succinate-supported mitochondrial respiration in KCl-based medium. Polymyxin B (100 µg/mL) caused a relatively low but sustained acceleration of respiration. Subsequent addition of TMPD and ascorbate, substrates donating electrons to cytochrome *c* and cytochrome *c* oxidase, caused a strong stimulation of respiration in the presence of the uncoupler FCCP, polymyxin B, and fusaricidins A+B at low concentrations (0.75 µg/mL). By contrast, the respiration in the presence of alamethicin, Ca^2+^, and fusaricidins A+B at high concentrations (2.5 and 5 µg/mL) was only slightly stimulated. Thus, FTCs, similarly to alamethicin, inhibited the TMPD- and ascorbate-supported respiration in a dose-dependent manner. One might suggest that this inhibition was due to the loss of cytochrome *c* by swollen mitochondria with the ruptured outer membrane. As it is shown in Figure 6B, the RS10 extract, fusaricidins A+B, and LI-F05a, indeed, induced a release of cytochrome *c* from liver mitochondria. The release was comparable to that caused by alamethicin at a high concentration and mPTP opening but much greater than that caused by polymyxin B at a high concentration.

These data indicated that FTCs accumulated in the IMM in a ΔΨ_m_-dependent way and created pores permeable for low-molecular-weight solutes (~750 Da), ions, and reactive oxygen species. A disturbance of ionic homeostasis caused a high-amplitude swelling of mitochondria, the rupture of the outer membrane, a release of cytochrome *c* from the intermembrane space, and the inhibition of respiration.

### 3.6. FTCs Cause Fast Permeabilization of Plasma Membrane to Low-Molecular-Weight Solutes

The permeabilization of the mitochondrial membrane by FTCs strongly depended on ΔΨ_m_ (see Figure 2and Figure 4 ). We examined whether FTCs could create pores in membranes with low transmembrane potential, such as the plasma membrane. The data presented in Table 2 demonstrate that there was a clear correlation between the disruption of ΔΨ_m_, inhibition of the motility, and the increase in the permeability of plasma membranes (PM) of boar spermatozoa to PI (~668 Da) caused by FTCs (fusaricidins A + B, LI-F05a, and the RS10 extract) at different concentrations.

The fact that the permeabilization of the plasma membrane occurred within one hour of incubation indicated that the process was not associated with mitochondria-dependent apoptosis in spermatozoa [27]. We explored whether fusaricidins and LI-Fs could make the plasma membrane of sperm cells permeable to ATP (507 Da) before it is oxidized by numerous intracellular ATPases.

**Figure 7:**
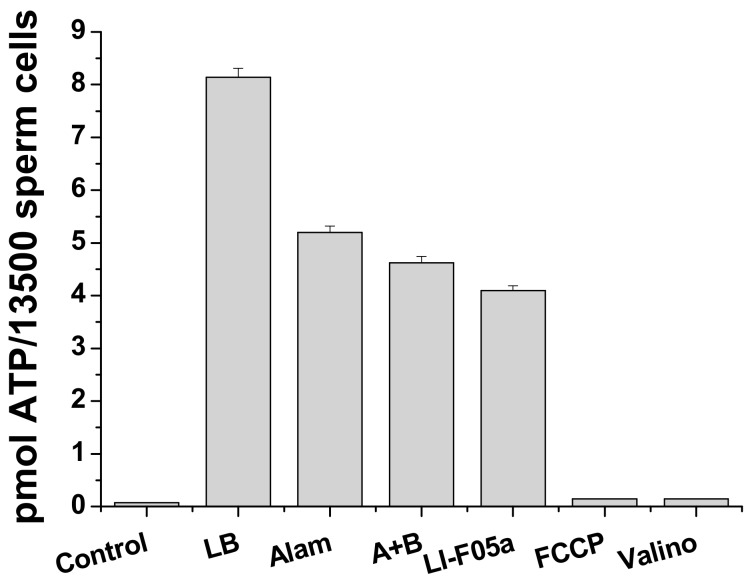
FTCs-induced ATP release from boar spermatozoa. Boar spermatozoa (in standard medium for sperm viability extension) were treated with lysis buffer (LB) or exposed to fusaricidins A + B (10.5 µg/mL), LI-F05a (5.625 µg/mL), alamethicin (12.5 µg/mL) (Alam), 500 nM FCCP, and valinomycin (25 ng/mL) (Valino) for 5 min and precipitated. Supernatants were examined for the presence of ATP using a luciferin luciferase kit, as described in Materials and Methods. The values in columns are the means ± S.E.M. (*n* = 3). The experiments were performed at 37 °C.

Figure 7 shows a massive release of ATP from boar spermatozoa caused by fusaricidins A+B (A + B) and LI-F05a during short incubation and sedimentation (5 + 5 min) in standard medium for sperm viability extension. At high concentrations, pure FTCs were almost as effective as the pore former alamethicin. The protonophore FCCP and the potassium carrier valinomycin (Valino) induced no ATP release. Hence, FTCs could cause fast permeabilization of the plasma membrane for solutes of ≥500–700 Da.

## 4. Discussion

*P. polymyxa* strains are the frequent colonizers of gypsum liners damaged by water and mold and containing substances toxic to mitochondria [28]. As well-known nitrogen-fixing bacteria, *P. polymyxa* can promote the growth of toxigenic mold in nitrogen-poor environments, such as gypsum liners. However, the occurrence of environmental *P. polymyxa* strains, capable of producing substances toxic to mammalian cells and representing a potential health hazard for humans and domestic animals, was poorly documented.

Earlier, we showed that FTCs made artificial black lipid membranes permeable for potassium ion and suggested that this could be a mechanism of toxicity of FTCs for mammalian cells [10]. Here, we first directly demonstrated that FTCs were capable of forming nonselective pores in mitochondrial and plasma membranes of mammals. The creation of pores causes a severe dysfunction of mitochondrial and plasma membranes, which is the basic mechanism of toxicity of fusaricidins and related substances to mammals (mouse, pig, and human) [1,10].

In the present work, no live animals were used for toxicity assessment. Nevertheless, the data obtained allow one to roughly estimate the potential toxicity of FTCs to mammals. Large specialized protein toxins (e.g., Botulinum neurotoxins, cytolysins, hemolysins, and aerolysins) from pathogenic *Clostridium*, *Streptococcus*, *Bacillus*, and *Aeromonas* bacteria are the most toxic among natural channel- and pore-forming agents [29,30]. The epsilon toxin from *Clostridium perfringens*, one of the most toxic biological substances (~400,000 mouse LD100/mg protein), permeabilizes cellular membranes of neuronal granular cells at a concentration as low as 10^−7^ M (~3 µg/mL) [30]. Melittin, a cationic 2.6 kDa peptide from the *Apis mellifera* bee venom, is hemolytic and cytotoxic starting from ~0.5 µM (1.3 µg/mL), with maximum activity at ~7 and 20 µM (18 and 52 µg/mL), respectively [31,32]. The peptaibols alamethicin and zervamicin permeabilize plasma membrane of mammalian somatic cells at concentrations of ~5 µM (10 µg/mL) [33]. Other peptides display the membrane activity and toxicity at comparable or higher concentrations [34]. Hence, FTCs, which were reported to be membrane-active and cytotoxic at concentrations of 0.5–10 µg/mL (0.5–10 µM) [10], should have comparable toxicity to mammals. What is more, positive charge, lipophilicity, and high thermal tolerance may increase the likelihood of the presence of FTCs in the indoor dust, prolonged contact of humans and animals with them, and, as a consequence, long-term cumulative effects on health [10].

Here, we showed that FTCs at high concentrations (5–10 µg/mL) created nonselective pores permeable to solutes of ~750 Da in mitochondrial membranes (see Figure 1 and Figure 5) and permeable to at least 500–700 Da compounds in the plasma membrane (see Figure 7 and Table 2). The permeabilization of membranes due to the interaction of the guanidine fragment with the regulatory sites of mPTP, as it was demonstrated for polycationic peptides BTM-P1 [15], p13II [16], and mastoparan [17], presumably, must be excluded. First, the mPTP antagonists EGTA and CsA were unable to prevent the permeabilization (see Figure 2). In contrast to BTM-P1 and p13II, which induced a low-conductance state of mPTP (<200 Da) [15,16], and mastoparan, which facilitated the full-size mPTP opening (~1500 Da) [17], FTCs created pores with an intermediate conductivity (see Figure 5). Second, FTCs permeabilized not only mitochondria but also plasma (see Figure 7 and Table 2) and black lipid membranes [10], which contained no mPTP components. Moreover, the fact that FTCs make the sperm cell plasma membrane permeable for ATP and PI within as less as 10 min (see Figure 7 and Table 2) demonstrates that FTCs affect the membrane directly, and permeabilization is not a consequence of the development of apoptotic or necrotic process [27].

**Figure 8:**
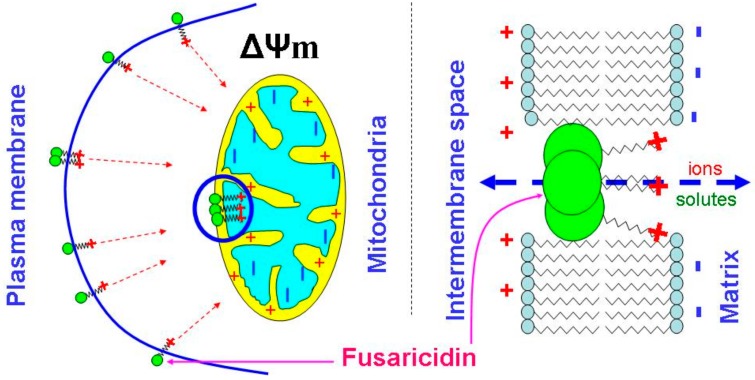
Probable role of the guanidine moiety in the ΔΨ_m_-dependent creation of fusaricidin pores in mitochondrial and plasma membranes.

One can suggest that the formation of fusaricidin pores requires the assembly of several molecules, as it takes place in the case of alamethicin [35]. However, the neutral head and the cationic tail of FTCs are not as long and rigid as molecules of peptaibols, e.g., alamethicin, which easily spans the lipid bilayer (~35 Å) [36]. Therefore, the formation of pores must depend on the capability of positively charged tails to pull uncharged heads into polarized membranes. Being incorporated into the membrane, bulky heads will disturb the membrane leaflet on the positive side, whereas tails, due to electrostatic repulsion, would disorganize the negative side of the membrane (Figure 8). The strong ΔΨ_m_-dependence of the effect of FTCs on mitochondrial swelling shows that the guanidine moiety is a critical element for the pore-forming activity and toxicity (see Figure 2 and Figure 4). Indeed, amino acid substitutions at positions 2, 3, and 5 in the ring (see Figure 3 and Table 1) [1,2,3,4,5] had no drastic effect on the pore-forming ability (see Figure 4, Figure 5, Figure 6 and Figure 7). By contrast, polymyxin B, which has a structure similar to that of FTCs but lacks the guanidine moiety at the end of the fatty acid tail, demonstrated ~100 times lower pore-forming ability in mitochondrial membranes (see Figure 6 and Figure A1). Positively charged guanidine should direct FTCs primarily into mitochondria, which possess the highest ΔΨ_m_ in the living cell. This can explain the fact that the activation of apoptotic death of murine fibroblasts L929 and porcine kidney epithelial PK-15 cells requires a concentration of fusaricidins 10 to 20 times lower than that necessary for the direct damage to the plasma membrane [10]. Presumably, the electrostatic interaction of guanidine with membranous phospholipids is also important for the incorporation of the toxin into the inner membrane, since mitochondrial swelling was more extensive in the sucrose-mannitol medium of a low ionic strength than in KCl or NaCl media (see Figure 1).

Alamethicin is widely used as a reference pore-forming compound in the studies of mPTP regulation and properties [22,37,38,39]. However, alamethicin-induced changes in the shape and functions of mitochondria are not identical to those caused by mPTP opening. Alamethicin created pores of a much larger size than mPTP (see Figure 5), which is associated with a stronger release of cytochrome *c* [38] and superoxide flashes [22], as well as with a larger amplitude of swelling, which, in turn, might more strongly interfere with the results of fluorescent and luminescent measurements (see Figure 5B). At the same time, FTCs created slightly smaller pores than mPTP and produced effects on mitochondrial respiration, swelling, superoxide flashes, and cytochrome *c* release similar to those caused by mPTP opening (see Figure 5; Figure 6).

## 5. Conclusions

The data obtained revealed the mechanism of toxicity of FTCs to mammals. Fusaricidins and LI-F compounds permeabilized the IMM and plasma membranes to ions and low-molecular-weight solutes (~750 Da) by the creation of nonselective pores. These pores were slightly smaller than the mPTP and produced similar effects on mitochondrial respiration, swelling, superoxide flashes, and the release of cytochrome *c*. Our results demonstrated that fusaricidins and LI-F compounds could be a useful instrument for studying the mPTP opening, which could be induced in various pathophysiologic states, including ischemia/reperfusion.

## Figures and Tables

**Table 1:** FTCs (fusaricidin-type compounds) found in HPLC fractions 1–4 of ethanol extracts of *P. polymyxa* strains RS10 and I/Sim.

Number of Toxic HPLC Fraction	Compound *	Mass Ion [M + H]^+^ m/z
1	Fusaricidin C	947.7
	Fusaricidin D	961.6
2	Fusaricidin A	883.7
	Fusaricidin B	897.6
3	LI-F05a	897.6
	LI-F06b/LI-F05b	911.6
	LI-F07b	945.5
	LI-F07a	931.6
4	LI-F06b/LI-F05b and LI-F07b	911.6 and 945.6
	LI-F08b	925.6
	Fusaricidin/LI-F **	960.5

Notes: * compounds are listed in order of decreasing signal intensity in a fraction; ** a newly identified fusaricidin/LI-F compound.

**Table 2:** Damage to boar spermatozoa caused by the RS10 extract and purified fusaricidins A + B and LI-F05a (HPLC fractions 2 and 3, respectively).

1-h Incubation	Motility	ΔΨ_m_ (JC1 Staining)	PM Permeability (PI Staining)
Control	≥60%	≥98%	≤5%
Ethanol (0.5%)	≥60%	≥98%	≤5%
RS10 (µg/mL) 50	1–2%	1–2%	>90%
25	~10%	5–10%	~60%
12.5	~40%	30–40%	30–40%
6.25	~50%	40–50%	≤20%
A+B (9.35 µg/mL)	1–2%	1–2%	~90%
LI-F05a (5.06 µg/mL)	5–10%	5–10%	≥70%

Note: the experiments were performed at 37 °C. ΔΨm: membrane potential; PM: plasma membrane; PI: propidium iodide; JC1: 5,5′,6,6′-tetrachloro-1,1′,3,3″-tetraethylbenzimidazolylcarbocyanine iodide.

## Abbreviations

ΔΨm	mitochondrial inner membrane potential
BSA	bovine serum albumin
CsA	cyclosporin A
EGTA	ethylene glycol-bis(2-aminoethylether)-N,N,N′,N′-tetraacetic acid
FCCP	carbonyl cyanide p-(trifluoromethoxy)phenyl-hydrazone
FTC	fusaricidin-type compound
HEPES	4-(2-hydroxyethyl)piperazine-1-ethanesulfonic acid
IMM	inner mitochondria membrane
JC-1	5,5′,6,6′-Tetrachloro-1,1´,3,3′’-tetraethylbenzimidazolylcarbocyanine iodide
MCLA	7-dihydro-2-methyl-6-(4-methoxyphenyl)imidazol[1,2-a]pyrazine-3-one
MDCL	MCLA-derived chemiluminescence
mPTP	mitochondrial permeability transition pore
PI	propidium iodide
PEG	polyethylene glycol
TMPD	N,N,N’N’-tetramethyl-p-phenylenediamide
TPP+	tetraphenylphosphonium chloride

## References

[1] Kurusu K., Ohba K., Arai T., Fukushima K. (1987). New peptide antibiotics LI-F03, F04, F05, F07, and F08, produced by Bacillus polymyxa. I. Isolation and characterization. J. Antibiot..

[2] Kajimura Y., Kaneda M., Fusaricidin A. (1996). A new depsipeptide antibiotic produced by Bacillus polymyxa KT-8. Taxonomy, fermentation, isolation, structure elucidation and biological activity. J. Antibiot..

[3] Kajimura Y., Kaneda M. (1997). Fusaricidins B, C, and D, new depsipeptide antibiotics produced by Bacillus polymyxa KT-8: Isolation, structure elucidation and biological activity. J. Antibiot..

[4] Choi S.K., Park S.Y., Kim R., Lee C.H., Kim J.F., Park S.H. (2008). Identification and functional analysis of the fusaricidin biosynthetic gene of Paenibacillus polymyxa E681. Biochem. Biophys. Res. Commun..

[5] Kuroda J., Fukai T., Konishi M., Uno J., Kurusu K., Nomura T. (2000). LI-F antibiotics, a family of antifungal cyclic depsipeptides produced by Bacillus polymyxa L-1129. Heterocycles.

[6] Cochrane S.A., Vederas J.C. (2016). Lipopeptides from Bacillus and Paenibacillus spp.: A gold mine of antibiotic candidates. Med. Res. Rev..

[7] Stawikowski M., Cudic P. (2009). Lipodepsipeptide antibiotic fusaricidin and its analogues. Total solid-phase and biological activity. Adv. Exp. Med. Biol..

[8] Beatty P.H., Jensen S.E. (2002). Paenibacillus polymyxa produces fusaricidin-type antifungal antibiotics active against Leptosphaeria maculans, the causative agent of blackleg disease of canola. Can. J. Microbiol..

[9] Lamsal K., Kim S.W., Kim Y.S., Lee Y.S. (2013). Biocontrol of late blight and plant growth promotion in tomato using rhizobacterial isolates. J. Microbiol. Biotechnol..

[10] Mikkola R., Andersson M.A., Grigoriev P., Heinonen M., Salkinoja-Salonen M.S. (2017). The toxic mode of action of cyclic lipodepsipeptide fusaricidins, produced by Paenibacillus polymyxa, toward mammalian cells. J. Appl. Microbiol..

[11] Ekman J.V., Kruglov A., Andersson M.A., Mikkola R., Raulio M., Salkinoja-Salonen M. (2012). Cereulide produced by Bacillus cereus increases the fitness of the producer organism in low-potassium environments. Microbiology.

[12] Chinopoulos C. (2018). Mitochondrial permeability transition pore: Back to the drawing board. Neurochem. Int..

[13] Grimm S., Brdiczka D. (2007). The permeability transition pore in cell death. Apoptosis.

[14] Xiong S., Mu T., Wang G., Jiang X. (2014). Mitochondria-mediated apoptosis in mammals. Protein Cell..

[15] Lemeshko V.V., Arias M., Orduz S. (2005). Mitochondria permeabilization by a novel polycation peptide BTM-P1. J. Biol. Chem..

[16] D’Agostino D.M., Ranzato L., Arrigoni G., Cavallari I., Belleudi F., Torrisi M.R., Silic-Benussi M., Ferro T., Petronilli V., Marin O. (2002). Mitochondrial alterations induced by the p13II protein of human T-cell leukemia virus type 1. Critical role of arginine residues. J. Biol. Chem..

[17] Pfeiffer D.R., Gudz T.I., Novgorodov S.A., Erdahl W.L. (1995). The peptide mastoparan is a potent facilitator of the mitochondrial permeability transition. J. Biol. Chem..

[18] Andersson M.A., Mikkola R., Rasimus S., Hoornstra D., Salin P., Rahkila R., Heikkinen M., Mattila S., Peltola J., Kalso S. (2010). Boar spermatozoa as a biosensor for detecting toxic substances in indoor dust and aerosols. Toxicol. In Vitro.

[19] Johnson D., Lardy H.A. (1967). Isolation of liver or kidney mitochondria. Methods Enzymol..

[20] Gornall A.G., Bardawill C.J., David M.M. (1949). Determination of serum proteins by means of the biuret reaction. J. Biol. Chem..

[21] Kambayashi Y., Ogino K. (2003). Reestimation of Cypridina luciferin analogs (MCLA) as a chemiluminescence probe to detect active oxygen species: Cautionary note for use of MCLA. J. Toxicol. Sci..

[22] Kharechkina E.S., Nikiforova A.B., Kruglov A.G. (2018). Pyridine nucleotides regulate the superoxide anion flash upon permeabilization of mitochondrial membranes: An MCLA-based study. Free Radic Biol Med.

[23] Kruglov A.G., Subbotina K.B., Saris N.E. (2008). Redox-cycling compounds can cause the permeabilization of mitochondrial membranes by mechanisms other than ROS production. Free Radic. Biol. Med..

[24] Kruglov A.G., Teplova V.V., Saris N.E. (2007). The effect of the lipophilic cation lucigenin on mitochondria depends on the site of its reduction. Biochem. Pharmacol..

[25] Andersson M.A., Jääskeläinen E.L., Shaheen R., Pirhonen T., Wijnands L.M., Salkinoja-Salonen M.S. (2004). Sperm bioassay for rapid detection of cereulide-producing Bacillus cereus in food and related environments. Int. J. Food Microbiol..

[26] Kuznetsov A.V., Javadov S., Saks V., Margreiter R., Grimm M. (2017). Synchronism in mitochondrial ROS flashes, membrane depolarization and calcium sparks in human carcinoma cells. Biochim. Biophys. Acta Bioenergy.

[27] Aitken R.J., Baker M.A. (2013). Causes and consequences of apoptosis in spermatozoa; contributions to infertility and impacts on development. Int. J. Dev. Biol..

[28] Andersson M.A., Nikulin M., Köljalg U., Andersson M.C., Rainey F., Reijula K., Hintikka E.L., Salkinoja-Salonen M. (1997). Bacteria, molds, and toxins in water-damaged building materials. Appl. Environ. Microbiol..

[29] Lai B., Agarwal R., Nelson L.D., Swaminathan S., London E. (2010). Low pH-induced pore formation by the T domain of botulinum toxin type A is dependent upon NaCl concentration. J. Membr. Biol..

[30] Lonchamp E., Dupont J.L., Wioland L., Courjaret R., Mbebi-Liegeois C., Jover E., Doussau F., Popoff M.R., Bossu J.L., de Barry J. (2010). Clostridium perfringens epsilon toxin targets granule cells in the mouse cerebellum and stimulates glutamate release. PLoS ONE.

[31] Liu S.I., Cheng H.H., Huang C.J., Chang H.C., Chen W.C., Chen I.S., Hsu S.S., Chang H.T., Huang J.K., Chen J.S. (2008). Melittin-induced [Ca2+]i increases and subsequent death in canine renal tubular cells. Hum. Exp. Toxicol..

[32] Pandey B.K., Ahmad A., Asthana N., Azmi S., Srivastava R.M., Srivastava S., Verma R., Vishwakarma A.L., Ghosh J.K. (2010). Cell-selective lysis by novel analogues of melittin against human red blood cells and Escherichia coli. Biochemistry.

[33] Weidema A.F., Kropacheva T.N., Raap J., Ypey D.L. (2007). Membrane permeabilization of a mammalian neuroendocrine cell type (PC12) by the channel-forming peptides zervamicin, alamethicin, and gramicidin. Chem. Biodivers..

[34] Kourie J.I., Shorthouse A.A. (2000). Properties of cytotoxic peptide-formed ion channels. Am. J. Physiol. Cell Physiol..

[35] Fox R.O., Richards F.M. (1982). A voltage-gated ion channel model inferred from the crystal structure of alamethicin at 1.5-A resolution. Nature.

[36] Chugh J.K., Wallace B.A. (2001). Peptaibols: Models for ion channels. Biochem. Soc. Trans..

[37] Broekemeier K.M., Iben J.R., LeVan E.G., Crouser E.D., Pfeiffer D.R. (2002). Pore formation and uncoupling initiate a Ca2+-independent degradation of mitochondrial phospholipids. Biochemistry.

[38] Petrosillo G., Ruggiero F.M., Pistolese M., Paradies G. (2004). Ca2+-induced reactive oxygen species production promotes cytochrome c release from rat liver mitochondria via mitochondrial permeability transition (MPT)-dependent and MPT-independent mechanisms: Role of cardiolipin. J. Biol. Chem..

[39] Korge P., John S.A., Calmettes G., Weiss J.N. (2017). Reactive oxygen species production induced by pore opening in cardiac mitochondria: The role of complex II. J. Biol. Chem..

